# Serous Borderline Tumor in Transgender Female-to-Male Individuals: A Case Report of Androgen Receptor-Positive Ovarian Cancer

**DOI:** 10.1155/2021/8861692

**Published:** 2021-06-04

**Authors:** Cristina Ferreira, João Fraga, Célia Antunes, Manuela Gonçalo, Paulo Donato

**Affiliations:** ^1^Department of Radiology, University Centre Hospitals of Coimbra (CHUC), 3000-075 Coimbra, Portugal; ^2^Department of Pathology, University Centre Hospitals of Coimbra (CHUC), 3000-075 Coimbra, Portugal

## Abstract

Ovarian cancer is the most fatal gynecologic malignancy. The incidence of ovarian cancer among female-to-male transsexuals receiving treatment with testosterone is unknown, and few cases have been reported in the literature. We report a recent case in our institution, a 23-year-old female-to-male transsexual patient who received testosterone supplementation. The patient underwent a pelvic magnetic resonance imaging to study an ovarian complex cyst that revealed the presence of a bilateral ovarian tumor with imaging features of borderline serous tumor. These masses were surgically removed and the pathology report confirmed the diagnosis associated with noninvasive peritoneal implants and the presence of numerous androgen receptors in the tumor cells. Although there is still insufficient data to validate a direct correlation between hormonotherapy and ovarian cancer in these patients, this case may reinforce previous reports on this association and highlights the relevance of radiological follow-up and bilateral salpingo-oophorectomy as part of gender reassignment surgery.

## 1. Introduction

It has been established that the acquisition of secondary sex characteristics of the other gender, based on sex steroids treatment, is fundamental for sex reassignment in transsexuals. However, an unresolved question is whether, in the long term, the administration of cross-sex hormones is safe [1].

Furthermore, it is believed to exist an underreporting of complications of cross-sex hormone therapy. This may occur because although the initial treatment is mainly administrated in specialized centers, complications on the long term are often seen in general practice, and they are then only occasionally reported in the scientific literature [1].

We present a case of a female-to-male (FTM) transgender patient who developed serous borderline ovarian tumor (SBOT) while taking testosterone injections.

SBOT is an intermediate grade of neoplasm between benign and malignant serous ovarian tumors [2]. Patients frequently refer abdominal distension or pain and serum CA-125 increases in half of patients [3, 4]. The more established treatment is bilateral salpingo-oophorectomy, hysterectomy, and omentectomy [5, 6]. Compared to malignant tumors, the prognosis of SBOT is far better, as the disease is usually confined to the ovary, having a >95% 10-year survival rate [7]. Nevertheless, 30% of SBOT are associated with peritoneal implants that can be noninvasive or invasive [8]. In the last case, survival rates of these patients fall considerably. Adjuvant chemotherapy can be offered to them, but there is no firm evidence of any survival benefits [9].

Currently, there is not enough evidence to support any recommendation to continue or stop the hormonal treatment. However, its duration probably increases the risk of development of hormone-related malignancies, especially in the case of long-term exposure.

Despite this, transsexuals are often reluctant in stopping hormone administration for fear that the secondary sex characteristics of the acquired sex will diminish [1].

## 2. Case Report

We report the case of a 23-year-old FTM transgender who had been taking testosterone since 2016 in order to gender reassignment. This patient suffered from asthma, treated with budesonide/formoterol fumarate dihydrate and cetirizine, without any other personal or family medical history. Moreover, this patient had never been pregnant and had not proceeded with any surgery.

On the ultrasound routine exam dated of June 2019, a complex cyst lesion was detected in the left ovary, with intracystic solid vegetation, measuring 3.0 cm. The patient was asymptomatic and did not stop the supplementation. Pelvic clinical examination was unremarkable. Blood tests showed high serum levels of CA-125 of 133 U/ml (institutional upper limit of normal, 27 U/ml). Thereby, the patient was presented to our department of radiology in order to perform a pelvic magnetic resonance imaging (MRI) study.

**Figure 1:**
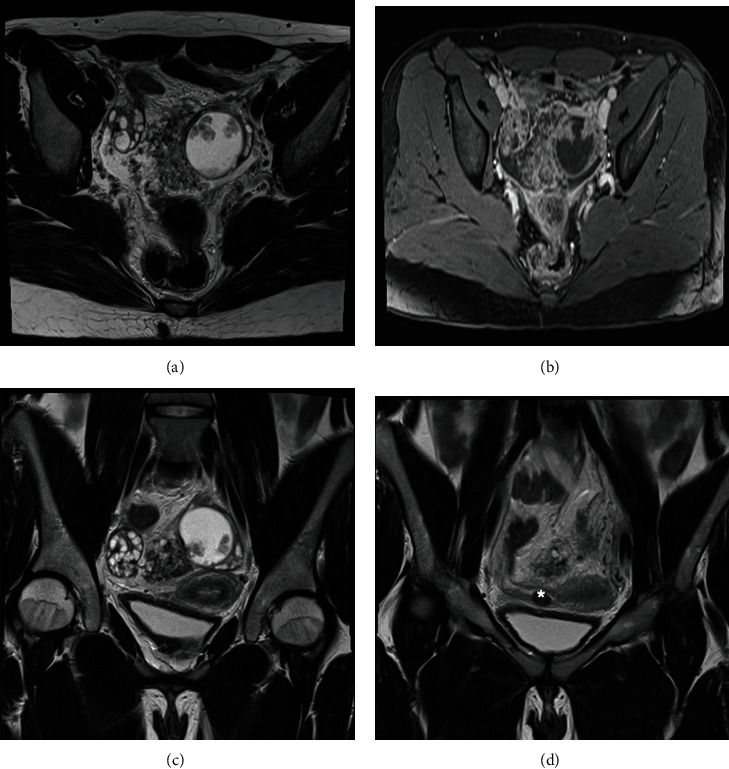
Pelvic MRI. T2-weighted axial image (a). Dynamic study (b). T2-weighted coronal image (c, d). There is a complex cystic lesion of the left ovary, with some solid papillary projections, which avidly enhance after contrast. Involving both ovaries there is another mixed mass, with the same characteristics of the previously described but arising from the right ovary. Both ovaries have multiple follicles, the right with polycystic appearance. Pedunculated leiomyoma (^∗^) of the right broad ligament. Small volume of peritoneal effusion in pelvic situation (1.5 T MRI machine).

**Figure 2:**
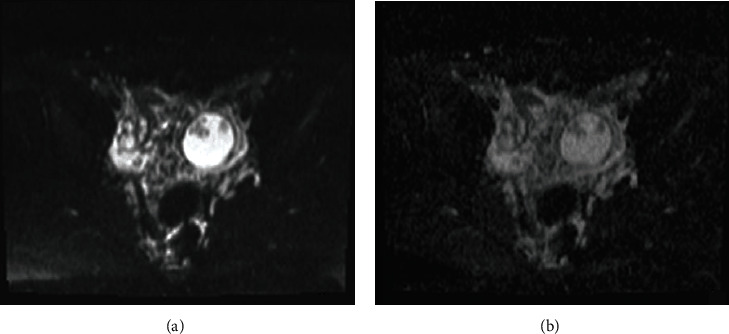
Axial diffusion-weighted (b1000) image (a) and diffusion coefficient map (b) show that papillary projections are nonrestricted on diffusion-weighted imaging.

MRI images were acquired using a 1.5 T MRI machine. Imaging parameters are provided in the figure legends (shown in Figures 1 and 2). The exam revealed a complex cystic lesion arising from the left ovarian and measuring 4.0 × 4.3 cm that displayed some solid intermediate to hypointense papillary projections, which avidly enhanced after contrast, but without diffusion restriction. There was another mixed mass, involving both ovaries, with the same characteristics of the previously described, but arising from the right ovary, being predominantly exophytic and measuring 10 × 5.8 cm. Both ovaries had also multiple follicles, the right with polycystic appearance. The diagnosis of bilateral serous borderline tumor has been suggested in the radiology report—papillary cystic variant in the left ovary and papillary superficial variant in the right ovary. The uterus was anteverted, with normal size and regular contours. A hypointense nodular lesion was identified at T1 and T2 sequences, next to the right uterine horn, measuring 1.2 cm, suggesting pedunculated leiomyoma of the right broad ligament. The endometrium was fine and regular. There was a small volume of peritoneal effusion in pelvic situation, and there was no evidence of suspected pelvic lymph nodes (shown in Figures 1 and 2).

**Figure 3:**
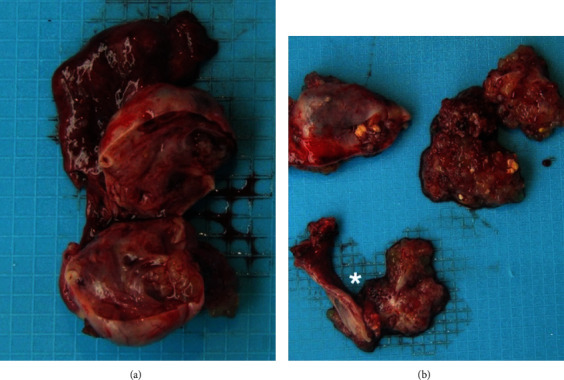
Macroscopic examination. Left ovary 6.5 × 3.5 × 1.5 cm, on the surface with friable nodular pink lesion, 4 × 5 × 1.4 cm. After opening, the ovary is replaced by a cystic formation, with translucent and filant content, with abundant lobulated nodules adhering to the inner surface, the largest with 1.5 cm in diameter, equally pink and friable. The cystic wall is thin and elastic (a). The contralateral annex (^∗^) shows a 6 cm horn and a 3 × 2.5 × 2 cm ovary, with a mostly pink and smooth surface, with an exophytic and lobulated lesion, with 2 × 1.5 cm. It is also identified a cystic formation with 1 cm, with a vegetation with 0.9 cm, which encompasses most of the cystic lesion (b).

In view of the imaging findings, the patient underwent surgery for resection of this mass, in October 2019. At the time of the laparotomy, left ovary was enlarged due to a complex cystic formation, measuring about 6.5 × 3.5 × 1.5 cm, that occupied the entire sac fundus of Douglas and was adherent to the anterior face to the bladder peritoneum. The right ovary had normal volume, presenting numerous lesions on its surface. Between the two ovaries, the lesion measured about 4.5 cm (shown in Figure 3). There was evidence of another adhesion between sigmoid colon and the tumor. Isolated nodules were also noted on the bladder fold and on serosal surface of the sigmoid colon, suggesting implants. Peritoneal serous effusion was also seen. Thus, a full surgical staging procedure included total hysterectomy, bilateral salpingo-oophorectomy, and multiple biopsies of the peritoneum. There were no other visible lesions in the remaining abdominal and pelvic cavities.

**Figure 4:**
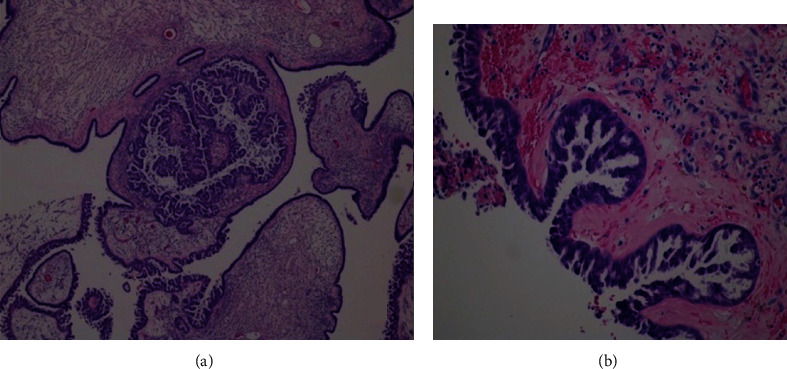
Pathological examination. Neoplasm with exophytic growth on the ovarian surface and, internally, in the macroscopically observed cystic areas. The lesion is mainly composed of papillae with large fibrovascular axes, with progressive and hierarchical branching, often with edema, hyaline sclerosis, and stromal calcifications. Tumor cells have eosinophilic cytoplasm and oval, hyperchromatic nuclei with mild atypia, with few mitotic figures being identified. There is no destructive invasion of stroma (H&E, 40x (a) and 100x (b)).

**Figure 5:**
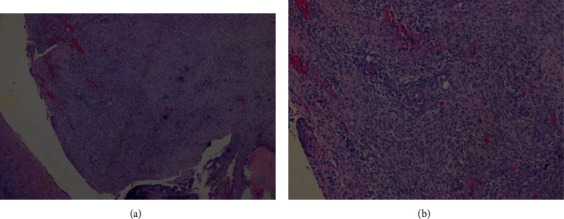
Autoimplants of the left ovary surface with dimensions ranging from 4 to 11 mm of larger axis, morphologically nodular, and consisting of abundant desmoplastic stroma, which involves small aggregates of neoplastic cells (H&E, 40x (a) and 100x (b)).

**Figure 6:**
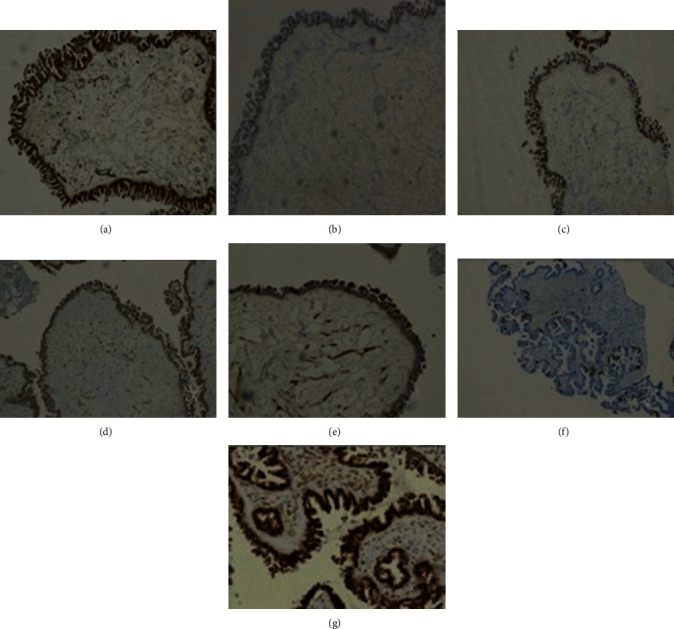
Immunohistochemistry study. Neoplastic cell positivity for WT1 (a), RE (80%) (b), RP (80%) (c), p53 wild-type labeling (faint 60%) (d), p16 (70%) (e), Ki-67 30% (f), and AR showing diffuse positivity (g) (100x).

The pathology report confirmed a bilateral borderline ovarian serous tumor with noninvasive desmoplastics implants (shown in Figures 4 and 5), and the immunohistochemical study revealed neoplastic cell positivity for WT1, RE (80%), RP (80%), p53 wild-type labeling (faint 60%), p16 (70%), and Ki-67 30% and AR (androgen receptors) diffuse positivity (shown in Figure 6).

After that, the patient was referred to the oncology department, for further management, and the hormone therapy was temporarily suspended until revaluation.

## 3. Discussion

Cross-sex hormone administration is relatively recent in medicine. Actually, its history started in the 1970s, and since then, few cases of hormone-dependent tumors have been reported in hormonally treated FTM transsexuals [1]. Only 4 cases of ovarian cancer in FTM transsexuals were reported in the scientific literature [10].

This case report describes a new case of a FTM transsexual patient who was under testosterone supplementation and who developed an ovarian cancer, namely, a serous borderline ovarian tumor.

Furthermore, this case report strengthens the role of radiology in the management of these patients. Indeed, ultrasound exam made it possible to detect the ovarian lesion. Additionally, MRI study helped the recognition of this ovarian tumor and its characterization, suggesting a serous borderline tumor, as well as the papillary cystic and papillary superficial subtypes. In fact, an abundance of intermediate to hyperintense papillary projections (edematous papillae) with hypointense internal branching (fibrous internal architecture of the papillary projections) and ovarian stroma preservation with a hypointense ovarian capsular margin, on T2-weighted imaging, are features strongly suggestive of this tumor [11]. When the high signal papillary projections are on the surface of the ovary without any cystic component, it suggests the diagnosis of the papillary superficial subtype. In the papillary cystic subtype, the papillary projections also occur within the cystic component [11]. Papillary projections are inconspicuous on T1WI. This tumor may restrict on diffusion-weighted imaging, but, due to its variability, this feature is not mandatory to stablish the diagnosis. Nevertheless, the enhancement after contrast is always present [11]. Moreover, the distinction between SBOT from benign and malignant tumors, in MRI exam, can be helped by the evaluation of the abundance and size of the papillary projections, with a direct correlation between the number and the dimension of these projections and the degree of malignant suspicion [11].

The final diagnosis was confirmed by the anatomopathological examination. In immunohistochemistry, WT1 is a marker of tissues derived from the middle mesoderm inner layer, combining positivity for estrogen receptors (ER) and progesterone receptors (RP), proving to be a neoplasm derived from the adnexal structures, so excluding the diagnosis of endometrioid carcinoma. High-grade serous carcinoma typically shows a p53 mutation with positivity in 100% of cells, as well as positivity to p16 and cell proliferation index (ki-67) around 100%, which was not observed in this clinical case report, thus reinforcing the diagnosis. Low-grade serous carcinoma can be immunohistochemically identical to the borderline serous tumor, but needs to have invasion criterion, namely, invasive implants. Finally, the tumor reported in this case showed abundant expression of androgen receptors, which also reinforces the diagnosis. It should be highlighted that androgen receptors are present in almost all epithelial ovarian cancers. Their function has been proposed to be associated with a growth stimulatory effect of testosterone and a possibly larger role in tumor progression.

From the above mentioned, we may consider a possible relationship between the testosterone supplementation and the development of the ovarian tumor in this patient, like few cases previously reported [12].

Studies have stated that short- and medium-term treatment of testosterone in FTM transsexuals is safe, but long-term safety has been difficult to guarantee [13].

Despite, in the current literature, there are no established special guidelines regarding the follow-up imaging recommendations for these patients neither defined dates for surgical procedures. These patients are, actually, advised to adhere to the routine cancer screening protocols as in nontransgender individuals, depending on the anatomic situation, according to birth-assigned sex [13].

Regarding to the surgical procedures to prevent gynecological cancer, it remains a controversial issue, because the oncological risk in FTM transsexuals, after the initiation of testosterone therapy, is still inconclusive and lacks power [13]. Some authors recommended salpingo-oophorectomy as the best treatment in FTM transsexuals, when they are fit for surgical sex reassignment, usually taking place 18–24 months after starting the testosterone administration [1]. Other authors do not support this recommendation, claiming the insufficient evidence linking exogenous androgens and ovarian malignancy [12].

On the other hand, there is also controversy on maintaining hormonal treatment in these patients. Hage et al. suggested that hormonal therapy should be stopped after the diagnosis of an androgen receptor-positive ovarian cancer [13]. However, Defreyne et al. argue that testosterone therapy should be uninterrupted lifelong after oophorectomy in order to maintain the secondary sex characteristics and to avoid hypogonadism symptoms [13].

Lastly, it is important to remember that transgender individuals commonly report avoidance of medical care for being afraid of discrimination [14]. On the other hand, there is a lack of well-informed and culturally competent health professionals in relation to these issues [15].

Also, taking into account all these controversies, by reporting this case, we reinforce the importance of the role of imaging during the follow-up of these patients, during hormonal therapy or while waiting for surgery, allowing the early detection and diagnosis of tumors, whose greater risk cannot be excluded.

## 4. Conclusion

This case report emphasizes that, despite the few described cases of ovarian tumors in transgender patients under testosterone therapy, we cannot underestimate its occurrence. Actually, this case was only detected by imaging studies, namely, with MRI, thus, reinforcing the importance of the role of imaging in monitoring these patients.
